# QTL mapping of thrips resistance in pepper

**DOI:** 10.1007/s00122-015-2558-1

**Published:** 2015-07-08

**Authors:** Awang Maharijaya, Ben Vosman, Greet Steenhuis-Broers, Koen Pelgrom, Agus Purwito, Richard G. F. Visser, Roeland E. Voorrips

**Affiliations:** Wageningen UR-Plant Breeding, Wageningen University and Research Center., P.O. Box 386, 6700 AJ Wageningen, The Netherlands; Bogor Agricultural University, Jalan Raya Darmaga, 16680 Bogor, Indonesia

## Abstract

*****Key message***:**

**A QTL for thrips resistance on pepper chromosome 6 was identified and validated. This QTL affects thrips larval development and explains 50** **% of the variation.**

**Abstract:**

Thrips is one of the most damaging pests in pepper (*Capsicum*). Resistance to thrips was identified in *Capsicum annuum.* This study was aimed at the elucidation of the genetic background of thrips resistance in *Capsicum* through QTL mapping. The QTL analysis was carried out for *Frankliniella occidentalis* resistance in an F_2_ population consisting of 196 plants derived from an interspecific cross between the highly resistant *C*. *annuum* AC 1979 as female parent and the highly susceptible *C. chinense* 4661 as male parent. Fifty-seven SSR, 109 AFLP, and 5 SNP markers were used to construct a genetic map with a total length of 1636 cM. Damage caused by larvae and the survival of first and second instar larval stages observed in a no-choice test were used as parameters of resistance. Interval mapping detected one QTL for each of these parameters, all co-localizing near the same marker on chromosome 6. Use of this marker as co-factor in a multiple-QTL mapping analysis failed to uncover any additional QTLs. This QTL explained about 50 % of the genetic variation, and the resistance allele of this QTL was inherited from the resistant parent. Thrips resistance was not linked to trichome density.

## Introduction

Pepper (*Capsicum*) production worldwide is constrained by thrips as one of the most damaging pests (Siemonsma and Piluek 1994). Adult thrips are about 1 mm long, and females are usually a bit larger than males. They belong to the insect order Thysanoptera. There are at least 16 species of thrips that attack *Capsicum* (Talekar 1991; Capinera 2001). Among these is *Frankliniella occidentalis*, which is the major species found on pepper in Europe (Tommasini and Maini 1995), and it has recently been found in Asia as well (Zhang et al. 2007). Thrips cause direct damage by feeding on pepper fruits, flowers, and leaves (Welter et al. 1990; Tommasini and Maini 1995; Shipp et al. 1998). Feeding of thrips on leaves may affect leaf size and carbon allocation in the plant (Welter et al. 1990; Shipp et al. 1998), reduce photosynthetic capacity (Tommasini and Maini 1995), and eventually reduce yield (Steiner 1990; Welter et al. 1990). Thrips feeding on pepper fruit causes bronzing and silvering of the fruit skin, thereby reducing market quality (Shipp et al. 1998). Thrips can also transmit several viruses, one of the most important being Tomato Spotted Wilt Virus (TSWV) (Ulman et al. 1992). This virus is acquired during the first and early second larval stage when there is a temporary association between mid-gut, visceral muscles, and salivary glands (Moritz et al. 2004). After that, the virus is transferred into a plant with the saliva of a feeding adult (Jones 2005).

Thrips management and control practices include chemical treatments, biological control, and integrated pest management. Unfortunately, they do not completely solve the problems caused by thrips (Reitz et al. 2003; Weintraub 2007). Thrips-resistant varieties would increase the effectiveness of thrips control. Resistance to thrips may also delay and reduce the transmission of viruses as was shown by Maris et al. (2003) for TSWV. Several pepper accessions have been found to carry resistance to thrips which may be exploited further to breed thrips-resistant varieties (Fery and Schalk 1991; Maris et al. 2003; Maharijaya et al. 2011, 2012).

Molecular marker linkage maps have been constructed for several *Capsicum* populations (Minamiyama et al. 2006; Yi et al. 2006; Barchi et al. 2007; Lee et al. 2009; Wu et al. 2009). These have been used to detect quantitative trait loci (QTLs) for plant development and fruit characteristics (Palloix et al. 2009; Borovsky and Paran 2011) and for resistance against pathogens, including anthracnose (*Colletotrichum* spp.) (Voorrips et al. 2004), *Phytophthora capsici* (Thabuis et al. 2004), and powdery mildew (Lefebvre et al. 2003). For resistance to thrips in pepper, a QTL has been identified by Syngenta Biotechnology Inc. on chromosome 5 (Linders et al. 2010). In other crops, QTLs for resistance to thrips were detected in cowpea (Muchero et al. 2010), potato (Galvez et al. 2005), common bean (Frei et al. 2005), and cabbage (Loptien 2013).

In earlier papers, we described the identification and characterisation of several sources of thrips resistance. We developed test methods to evaluate plant resistance to thrips and showed that these in vitro tests (detached leaf assays) correlate well with greenhouse tests based on damage scores (Maharijaya et al. 2011). The effect of resistance in pepper on thrips reproduction and development was studied using three highly resistant, three medium resistant, and three susceptible accessions. Resistance factors in leaves of resistant pepper accessions were shown to have significant effects, mostly on the larval stages. These factors completely blocked the development of L_1_ larvae to the L_2_ stage on the resistant accessions (Maharijaya et al. 2012).

Our current study was aimed at elucidating the genetic basis of the resistance to thrips that we identified earlier in *Capsicum annuum* AC 1979 (Maharijaya et al. 2011, 2012) through a QTL mapping approach. Since the resistant parent of our population was the same as the one used by Linders et al. (2010) we compared the mapping results. The presence of trichomes has been implicated in resistance against the thrips *Scirtothrips dorsalis* (Yadwad et al. 2008) in pepper, therefore we included this trait in our study as well.

## Materials and methods

### Plant material

A mapping population consisting of 196 F_2_ plants was developed from a cross between *C. annuum* AC 1979 as female parent and *C. chinense* 4661 as male parent. The parents were chosen based on evaluation results for resistance against two thrips species, *F*. *occidentalis* and *Thrips parvispinus* using several different resistance tests (Maharijaya et al. 2011, 2012). *Capsicum annuum* AC 1979 was highly resistant while *C. chinense* 4661 was very susceptible in these tests. Apart from the contrasting resistance levels the parents also differ in leaf characteristics (Wahyuni et al. 2011) including trichome density: *C. annuum* AC 1979 does not have trichomes while *C. chinense* 4661 is densely covered with trichomes. Both accessions were obtained from the Centre for Genetic Resources, the Netherlands. An interspecific cross was used to obtain a sufficient level of polymorphism in the F_2_ population.

The F_2_ population was grown together with two first-generation inbred lines, obtained by self-pollination of the two parental plants, and with cuttings of the F_1_ in a glasshouse at Wageningen University and Research Centre, the Netherlands. From 4 F_2_ plants that were heterozygous for marker Isotig18917-234 (close to the QTL maximum) F_3_ lines were obtained by selfing. A total of 41 F_3_ plants were used in this study.

The plants were maintained in a glasshouse at 25 °C, 16/8 h day/night without pesticide application. Pests were controlled biologically using predator organisms according to standard Dutch pepper cultivation practices. Seeds were sown in rockwool plugs in trays; seedlings were transplanted onto rockwool 5 weeks after germination. The F_3_ plants were grown under the same conditions but in a later year.

### Thrips

A *F. occidentalis* population was collected from thrips-infested *Arabidopsis thaliana* plants in a greenhouse of Wageningen UR (Wageningen, the Netherlands). After confirmation of the collected thrips as *F. occidentalis* a population was developed and maintained by rearing female thrips on small cucumber fruits in a climate chamber at 25 °C, 16/8 h day/night. Thrips larvae (L_1_ stage) were obtained by allowing thrips to lay eggs in small cucumber fruits for 1 day, after which the adult thrips were brushed off and fruits were kept at 25 °C for 4 days, when the larvae emerged (Mollema et al. 1993). The number of synchronized larvae was sufficient to infest a complete replication of the resistance test in 1 day.

### Resistance test

Five newly emerged *F. occidentalis* L_1_ larvae were placed on a single fresh fully opened leaf that was placed with the abaxial side downwards in a sterile 50 × 9 mm petri dish with lid (BD Falcon^®^). We used the third to sixth fully opened leaf counting from the top of the stem, taken from plants between 6 and 9 weeks after transplanting. Leaves and larvae were incubated in a climate chamber at 25 °C, 16 h light, and 70 % RH.

Damage caused by larvae was scored after 2 days using a visual scale ranging from 0 (no damage) to 3 (severe damage) as described in Maharijaya et al. (2011). Development of L_1_ larvae into the L_2_ stage was assessed by counting the number of L_2_ larvae and dividing this by the total number of larvae placed on the leaf. The transition from larval stage L_1_ to L_2_ was determined by the presence of skin tissue that remained on the leaf disk after molting, which can be seen under a stereo microscope. Development of L_2_ larvae was assessed by counting the number of pre-pupae divided by the original number of L_1_ larvae. Pre-pupae can be recognized by the presence of short wing sheaths. Leaves were replaced by fresh ones every 3 days until all larvae had died or reached the pre-pupa stage; this required incubation and observation up to 8 days. These two parameters are referred to as “survival to L_2_” and “survival to pre-pupa,” respectively.

For the F_2_ population experiment, each replication consisted of one petri dish per F_2_ plant, three dishes for each parental inbred, and two dishes of the F_1_. The complete F_2_ test consisted of five replications. The F_3_ lines experiment consisted of four replications, each with one dish per F_3_ plant. In both tests, each replication was started on a single day with approximately 1 week intervals.

### Trichome density

Trichome density was scored according to a visual scale: 0 (<50 cm^−2^), 1 (50–100 cm^−2^), 2 (100–200 cm^−2^), and 3 (>200 cm^−2^) at the region near the veins and midrib on the abaxial side of a fully developed leaf. Observations of trichome density were done at three different plant stages: early vegetative stage (3 weeks after transplanting), vegetative stage (6 weeks after transplanting), and reproductive stage (9 weeks after transplanting). Observations were performed on the third to sixth fully expanded leaf, counting from the top of the stem.

### Statistical analysis

#### F_2_ population experiment

Means for each F_2_ plant, the parental inbreds and the F_1_ were obtained by ANOVA analysis with the five replications of the resistance test as blocks, after transforming the fraction survival to L_2_ and pre-pupa stages as *y* = arcsine (sqrt(*x*)) in order to stabilize variances. Pearson correlation coefficients were calculated for the three parameters observed in the resistance test and leaf trichome densities, based on the means of F_2_ individuals. Broad-sense heritability was estimated for all parameters according to Allard (1999) using the formula: Heritability (*h*^2^) = (*σ*^2^F_2_ − (σ^2^F_1_ + σ^2^P_R_ + σ^2^P_S_)/3)/(σ^2^F_2_), where σ^2^F_2_, σ^2^F_1_, σ^2^P_R_, and σ^2^P_S_ are the variances of the F_2_, F_1_, resistant, and susceptible parent, respectively.

#### F_3_ lines experiment

Like in the F_2_ experiment, means for each F_3_ plant were obtained by ANOVA analysis with the four replications of the resistance test as blocks, after transforming the fraction survival to L_2_ and pre-pupa stages as *y* = arcsine (sqrt(*x*)) in order to stabilize variances. The means per plant were treated as response variable in a linear regression model with F_3_ line and marker score (0 = homozygous *annuum* allele, 1 = heterozygous, and 2 = homozygous *chinense* allele) as regressors.

### Molecular markers and linkage map

The KingFisher^®^ (www.thermo.com) device was used with the AGOWA mag^®^ Maxi DNA Isolation Kit (www.agowa.de) for isolating genomic DNA of the F_2_ and F_3_ individuals, F_1_, and parents. AFLP (Amplified Fragment Length Polymorphism) markers as described by Vos et al. (1995) were detected using combinations of *Eco*RI and *Mse*I or *Pst*I and *Mse*I primers with two selective nucleotides for *Pst*I and three selective nucleotides for *Eco*RI. The pre-amplification primers were E01, P00, and M02. Fifteen primers combination were used: P17-M39, P17-M32, P14-M50, P14-M49, P14-M48, P14-M41, P11-M61, P11-M48, E38-M49, E36-M48, E35-M58, E35-M49, E35-M48, E34-M48, and E32-M49 [primer sequences as in Keygene (2004)]. The *Pst*I and *Eco*RI primers were labeled with fluorescent dyes IRD700 and IRD 800 (Li-Cor, Lincoln, USA). The AFLP products were separated and visualized on a 6 % denaturing polyacrylamide gel using a Li-Cor^®^ sequencer. AFLP data were scored using Quantar software (Keygene^®^). Polymorphic bands were scored co-dominantly when there was a distinct difference in intensities between putatively homozygous and heterozygous bands.

Fifty-seven primer pairs were used to amplify simple sequence repeat (SSR) markers, which were used to assign the linkage groups obtained to pepper chromosomes based on published maps (Yi et al. 2006; Lee et al. 2009; Wu et al. 2009) and an unpublished map from INRA (Institut National de La Recherche Agronomique, France; personal communication, Dr. A. Palloix) (Table 1). The PCR mix for SSR markers contained 5 µl of 50 ng genomic DNA, 0.25 µl 1 M each of forward and reverse primer, 0.4 µl dNTP, 1 µl LC Green^®^ (Idaho Technology), 0.1 µL Phire™ Hot Start DNA Polymerase (Finnzymes^®^), 2 µL buffer, and 5 µl MQ. The solution was overlaid with 20 µL of mineral oil. The thermal cycling condition were set as follows: incubation at 94 °C for 2 min, 40 cycles of 94 °C for 60 s, 60 °C for 60 s, 72 °C for 60 s, followed by 5 min 72 °C extension, and hold at 4 °C. The PCR products were analyzed with the LightScanner^®^ system (Idaho Technology) using melting temperature from 60 to 95 °C at the default melting rate (0.1 °C s^−1^). LightScanner^®^ analysis software was used to normalize the curves and to score them as heterozygote or one of the two homozygotes. In cases where the heterozygote patterns could not be well discriminated from one of the homozygotes the marker was scored dominantly. Four SNP markers [LM_2001, LM_2002, LM_2004, LM_2006, developed by Linders et al. (2010)] were used as reference for the position of the QTL for thrips resistance identified chromosome 5 by this group. One SNP marker (Isotig18917-234, personal communication, Dr. A. Palloix) close to the maximum of the thrips resistance QTL on chromosome 6 identified in the present study was used as reference for this QTL. The PCR protocol, visualization, and scoring methods for these markers were the same as those for SSR primers. PCR primers for the 57 SSR and 5 SNP markers are listed in Table 1.

**Table 1:** List of chromosome assignments and primers for SSR and SNP markers

	Markers	Chr.^a^	Forward primer (5′–3′)	Reverse primer (5′–3′)
1	Epms 725	1	TTGAATCGTTGAAGCCCATT	ATCTGAAGCTGGGCTCCTTT
2	Hpms 1-41	1	GGGTATCATCCGTTGAAAGTTAGG	CAAGAGGTATCACAACATGAGAGG
3	Hpms 1-281	1	TGAGGCAGTGGTATGGTCTGC	CCCGAGTTCGTCTGCCAATAG
4	Gpms 169	2	TCGAACAAATGGGTCATGTG	GATGAGGGTCCTGTGCTACC
5	Gpms 37	2	ATTTGTATATTATTTCTTGGCCTTG	TGAACTACCCAATTCCAGCC
6	Hpms E073	3	TTATTCAGGCCCACTTATCGAA	CAGCAGCCAAATTCTTGATTTC
7	Hpms E008	3	CCCCTTAACTTTTAATTCTAGATCTGC	TCGTTGTTCCTCCATCACCTCA
8	Gpms 198	3	AGCTTTAGACAGTGTCTGCGTG	TGATGATAAATTGCCTTCCG
9	Epms 386	3	ACGCCAAGAAAATCATCTCC	CCATTGCTGAAGAAAATGGG
10	Hpms E122	3	GCAATGGCTCAGGTCTCCATCT	TGTCGCCCTTTAATGCAAAACC
11	Gpms 93	3	ATCCTTGGCGTATTTTGCAC	TTCACTTTGCACACAGGCTT
12	HpmsAT2 14	4	TTTAGGGTTTCCAACTCTTCTTCC	CTAACCCCACCAAGCAAAACAC
13	Hpms 1-165	4	GGCTATTTCCGACAAACCCTCAG	CCATTGGTGTTTTCACTGTTGTG
14	Hpms E099	4	CAATCATTGCCACCTTATTTTTGC	TCACAAGGGGTTGATGGAAATG
15	Hpms E055	4	GGCCGCTTAAAGTTGTTCAAGG	TGTGGCTAGCGGTGTTATGCAC
16	Hpms E049	4	CACTCCAACAGCAGCAGCAAAC	CCTTGCCGATGTTGAAGCTTTT
17	Hpms E085	4	TGCCCAAATATCAGTCAAGCTCA	TGGTTGTTGTTCTCATGGTGGTG
18	Hpms E111	4	CCATCATTTCTCCCCAATTCCA	GAGAGCAGAAGAAGGGGTGGTG
19	Hpms E116	5	CATCTCTCCGTTGAATCTATTTCC	ACGGTCATCCATTAGAACCGTA
20	Hpms 2-45	5	CGAAAGGTAGTTTTGGGCCTTTG	TGGGCCCAATATGCTTAAGAGC
21	Gpms 165	5	TGAACAATAATAATTGACAGGACAG	AGCCTCGCAGTTTGTTCTTAC
22	Hpms 2-23	5	CCCTCGGCTCAGGATAAATACC	CCCAGACTCCCACTTTGTG
23	Hpms E015	5	TTGTGAGGGTTTGACACTGGGA	CCGAGCTCGATGAGGATGAACT
24	Hpms E014	6	CTTTGGAACATTTCTTTGGGGG	GCGGACGTAGCAGTAGGTTTGG
25	Hpms E088	6	GCAAATGGTTCCCTAAACTGCTT	GCTCTCCGTTTCCGATGTGATT
26	Hpms E078	6	TTTGTGAAGAAGCAACCGGTGA	TGTGAGGAAGAAAGTGCGAAGG
27	Hpms 1-5	6	CCAAACGAACCGATGAACACTC	GACAATGTTGAAAAAGGTGGAAGAC
28	HpmsAT2-20	6	TGCACTGTCTTGTGTTAAAATGACG	AAAATTGCACAAATATGGCTGCTG
29	HpmsE113	6	CCCTAAAGCTCGAGAAATTGAAGC	GAATGCTGTTGCTGGGGTTGTT
30	Epms 376	6	ACCCACCTTCATCAACAACC	ATTTGTGGCTTTTCGAAACG
31	Hpms E068	7	TGTTCCTTTTGTTGTTACCTTTTG	CGTCTAGGAATGGAAGAAGAGC
32	Hpms E057	7	ACCCACTCCCTCTCCTCTTTGG	GCAGTGGAAAAACAGTCCTGTGG
33	Hpms 1-227	7	CGTGGCTTCAAGTATGGACTGC	GGGGCGGAACTTTTCTTATCC
34	Epms 342	8	CTGGTAGTTGCAAGAGTAGATCG	ATGATCTTTGACGACGAGGG
35	Hpms E115	1/8	TCATCTCATAGCCTGCCCCCTA	CCACTTGAAGAAGCCATGACCA
36	Hpms 1-148	1/8	GGCGGAGAAGAACTAGACGATTAGC	CCACCCATTCCACATAGACG
37	Hpms E004	1/8	TGGGAAGAGAAATTGTGAAAGCA	CAATGCCAACAATGGCATCCTA
38	Epms 310	8	TGGGAAGAGAAATTGTGAAAGC	AGGAAACATGGTTCAATGCC
39	Gpms 194	9	AGGTGGCAGTTGAGGCTAAG	GTTCTAGGTCTTTGCCCTGG
40	Hpms 1-3	9	TGGGAAATAGGATGCGCTAAACC	AACTTTAAGACTCAAAATCCATAACC
41	Hpms E051	9	TGGCCAGCTTCACACAGAGGTA	TGTCACAATATTGGAGGCCAGAA
42	Epms 419	9	TTCAGGTGCAGGTATCATCG	GGGTACTTGTCCATTTATCCAG
43	Hpms E143	9	CCATTCAGCTAGGGTTCAGTCCA	CGACCAAATCGAATCTTCGTGA
44	Hpms E013	10	GCGCCAAGTGAGTTGAATTGAT	CACCAATCCGCTTGCTGTTGTA
45	Hpms E059	10	GCAAGGACGCAGTCGTTAGACA	CCGCCTGTGCTGAATTGTTTAG
46	Hpms 2-21	10	TTTTTCAATTGATGCATGACCGATA	CATGTCATTTTGTCATTGATTTGG
47	Hpms E065	10	TGAAATAGGCCAATCCCTTTGC	ATTCCCTGGGATTCCTGCATTA
48	Hpms E031	10	CCCTAAATCAACCCCAAATTCAA	CCCCCATTACCTGACTGCAAAA
49	Hpms E096	10	CGGGTCAAACAAAAACCGAAGT	GCTTGTGGTTGAGCTCGCTCTT
50	Gpms 159	10	AAGAACATGAGGAACTTTAACCATG	TTCACCCTTCTCCGACTCC
51	Epms 561	11	ATTGGACTTCAAATTTGGCC	AAACCAAAATCAGCATTAAAATATAAAC
52	Epms 410	11	GGAAACTAAACACACTTTCTCTCTC	ACTGGACGCCAGTTTGATTC
53	Epms 391	11	TTTCTTCTCTGGCCCTTTTG	ACGCCTATTGCGAATTTCAG
54	Hpms 2-2	11	GCAAGGATGCTTAGTTGGGTGTC	TCCCAAAATTACCTTGCAGCAC
55	Hpms E094	12	CCAGTTGAGAGCTGCTGCAAAA	CACCAACAAAACAAAGGCCACA
56	Hpms E128	12	TGGATCCCAAAAGACTCAGAACA	TATTTCCCTCAGTCGAGGTCGT
57	Hpms E064	12	CCCTCCTTTTACCTCGTCAAAAA	ATGCCAAGGAGCAATGAGAACC
58	LM_2001	5	CTTTGGAGGTAGCGGTATG	CAACAAACGAACCACAATG
59	LM_2002	5	CCCGTTTACAAGCAAAGAG	GACCCCTGAAGAACCTCTC
60	LM_2004	5	TGTAGGATTACAAGAACATTATCG	GCGAGCTATTACACCGAAG
61	LM_2006	5	TCGGCCTGACTAGTATTGAC	CGGGTACCAGATGTAGGG
62	Isotig18917-234	6	ACTAGTAAGAGCAGGGGTG	TCAATAGATCCAAATGCAGATTGAAC

Putative chromosome positions and primer sequences of the Hpms markers are based on Lee et al. (2009) and Yi et al. (2006); of the Gpms and Epms markers on an unpublished Institut National de La Recherche Agronomique (INRA, France) map (personal communication, Dr. A. Palloix) and Wu et al. (2009); of the LM markers on Linders et al. (2010); of SNP marker Isotig18917-234 were communicated by Dr A. Palloix

^a^Putative chromosome position

A linkage map was constructed using JoinMap 4.1 software (Van Ooijen 2006). Markers with more than about 40 missing values were discarded. Groups of markers of a more or less constant composition over a range of LOD values were used as a starting point to create linkage groups. Where multiple linkage groups were found with SSR markers known to reside on the same pepper chromosome an attempt was made to combine the markers into one linkage group. Mapping within linkage groups was carried out with the regression algorithm and a maximum jump level of 5. The final result was obtained by deleting markers that did not fit well as judged by the nearest neighbor stress or the mean Chi square contribution.

### QTL mapping

Potential QTLs for damage, larval survival, and trichome density were identified using the MapQTL 6.0 package (Van Ooijen 2009). First, interval mapping analysis was performed to find regions with potential QTL effects. Second, co-dominant markers in these regions were used as co-factors in multiple-QTL mapping (MQM). Significance thresholds of log of odds (LOD) corresponding to a genome-wide confidence level of *P* < 0.05 were determined for each trait using the permutation test of MapQTL 6.0 with 1000 iterations. The QTL graphs were prepared with MapChart 2.3 (Voorrips 2002).

## Results

### Thrips resistance in the F_2_ population

**Fig. 1:**
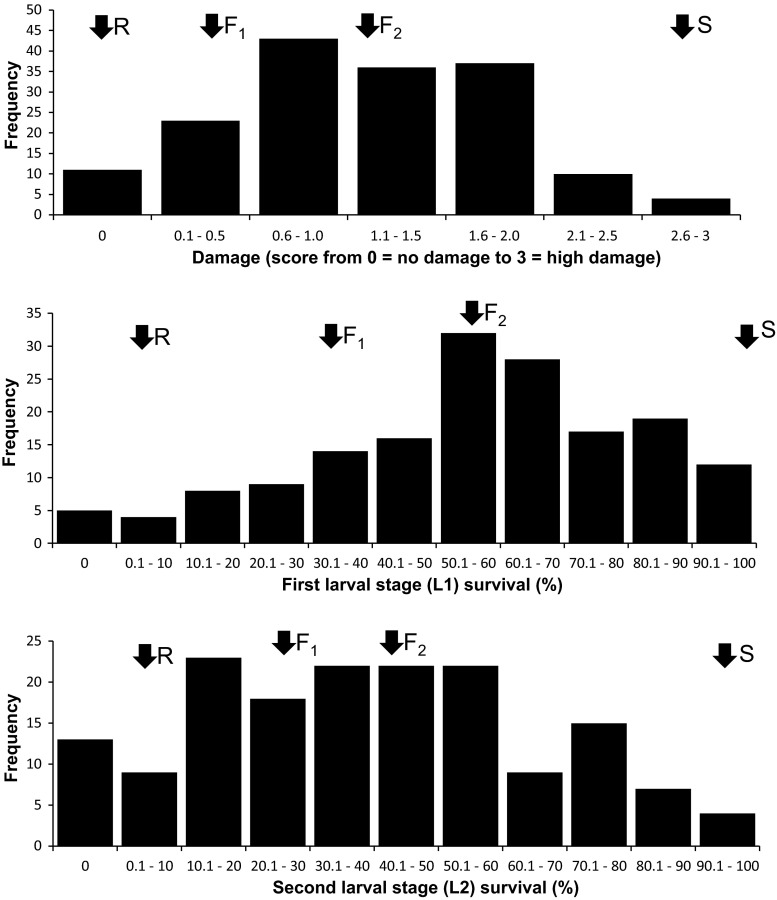
Frequency distributions for overall damage caused by first instar larva, survival to L_2_ (second larval stage), and survival to pre-pupa in F_2_ population from a cross between resistant and susceptible accessions of pepper. *Arrows* indicate the approximate means of the resistant parent (*R*), susceptible parent (*S*), F_1_ and F_2_ population

The F_2_ population showed a continuous variation for damage caused by larvae, for survival from L_1_ to L_2_, and for survival to pre-pupa (Fig. 1). Frequency distributions of damage and survival to pre-pupa were skewed toward the resistant parent, while for survival to L_2_ it was skewed toward the susceptible parent. In all replicates of the resistant parent, the damage was 0 and the survival to L_2_ and survival to pre-pupa was very low, while all replicates of the susceptible parent exhibited significant feeding damage and very high rates of survival to L_2_ and to pre-pupa. The broad-sense heritability of all parameters scored in the laboratory tests with *F. occidentalis* was high (Table 2).

**Table 2:** Values of resistance-related traits for parents, F_1_ and F_2_ plants after infestation with newly emerged L_1_ larvae of *Frankliniella occidentalis*

	Damage^b^	Survival to L_2_^c^	Survival to pre-pupa^d^
Resistant parent	0.00 ± 0.00^a^	0.20 ± 0.12	0.20 ± 0.12
Susceptible parent	2.73 ± 0.04	1.57 ± 0.00	1.36 ± 0.20
F_1_	0.40 ± 0.28	0.60 ± 0.06	0.55 ± 0.12
F_2_	1.16 ± 0.69	0.88 ± 0.38	0.66 ± 0.38
Heritability^e^	0.94	0.96	0.93

^a^Mean ± standard deviation

^b^Score of relative damage caused by L_1_ larvae of *F. occidentalis* at 2 days after infestation: 0 (no damage) to 3 (severe damage)

^c^arcsine(sqrt(*x*)) transformation of fraction of L_1_ larvae that survived to L_2_ stage

^d^arcsine(sqrt(*x*)) transformation of fraction of L_1_ larvae that survived to pre-pupa stage

^e^Broad sense heritability

Damage caused by larvae, survival of L_1_ to L_2_ and survival to pre-pupa were highly correlated with coefficients 0.68–0.78 (*P* < 0.001). None of the parameters scored in the resistance tests were significantly correlated with trichome density (Table 3).

**Table 3:** Spearman rank correlation coefficients and significance between all parameters scored in the F_2_ population

	Survival to L_2_^a^	Survival to pre-pupa^b^	Leaf trichome density		
			Early vegetative	Late vegetative	Reproductive
Damage caused by larva	0.68*	0.72*	0.13	0.11	0.12
Survival to L_2_^a^		0.78*	0.10	0.09	0.09
Survival to pre-pupa^b^			0.08	0.09	0.09
Leaf trichome density					
Early vegetative				0.86*	0.71*
Late vegetative					0.83*

Leaf trichome density was measured in three life stages of the plant

* Indicates significance *P* < 0.001

^a^arcsine(sqrt(*x*)) of fraction of L_1_ larvae that survived to L_2_ stage

^b^arcsine(sqrt(*x*)) of fraction of L_1_ larvae that survived to pre-pupa stage

### Linkage map

A linkage map was constructed consisting of 22 linkage groups. The linkage groups varied in length between 16.5 and 197.8 cM, with a total length of 1636.2 cM. The total map included 171 markers (57 SSR, 109 AFLP, and 5 SNP), of which 88 (51.5 %) were scored co-dominantly.

Linkage groups were assigned to pepper chromosomes based on SSR anchor markers. Seven chromosomes (1, 2, 6, 7, 8, 9, and 11) had only one linkage group assigned, while the other five had two or in one case (chromosome 3) three linkage groups assigned. Four linkage groups consisting of a total of 20 AFLPs and spanning 205.1 cM could not be assigned to chromosomes. Four markers (LM_2001, LM_2002, LM_2004, and LM_2006) described by Linders et al. (2010) as mapping to chromosome 5 were confirmed to map on that chromosome.

### QTL mapping

**Fig. 2:**
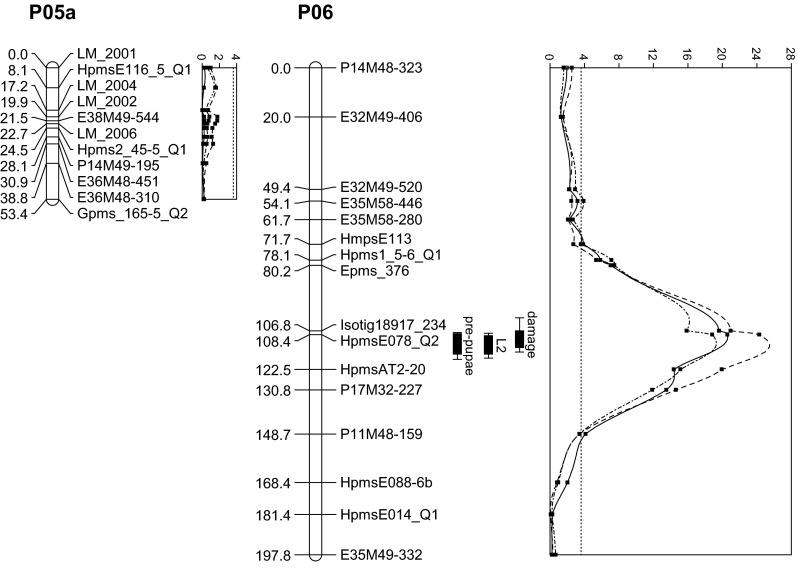
LOD profiles and 1-LOD and 2-LOD support intervals for resistance QTLs on chromosomes 5 and 6. *Solid*, *dashed*, and *dot-dashed lines* represent the profiles for damage, survival to L_2_, and survival to pre-pupae, respectively, after inoculation with newly emerged L_1_ larvae of *F. occidentalis.* The *dotted line* at LOD 3.6 represents the LOD threshold. On chromosome 5 no QTLs were detected for these traits

Interval mapping of damage, survival to L_2_, and survival to pre-pupa all resulted in the detection of the same, highly significant QTL on chromosome 6 (P06, Fig. 2). MQM mapping using the marker nearest to the top of the three LOD profiles (Hpms078) as co-factor failed to reveal any additional QTLs. In particular, no QTL signal was found on chromosome 5 at the four markers mentioned by Linders et al. (2010) to target a QTL for thrips resistance (Fig. 2). The LOD scores at marker Hpms078 were 20.6, 24.3, and 18.8, with an explained phenotypic variance of 43.9, 49.4, and 41.1 % for damage, survival of L_1_ to L_2_, and survival to pre-pupa, respectively (Table 4); a LOD threshold of 3.6 corresponding to a genome-wide *P* = 0.05 was estimated by a 1000-fold permutation test for all three traits. Since the heritabilities of damage, survival to L_2_ and survival to pre-pupa were 0.94, 0.96, and 0.93 (Table 2), the QTL explained 46.7, 51.5, and 44.2 %, respectively, of the genetic variance in the F_2_ for the three traits. The resistance allele of this QTL was inherited from the resistant parent. The dominance effect of the QTL was small in comparison with the additive effect, with susceptibility being partially dominant over resistance (Table 4).

**Table 4:** QTL effects for resistance-related traits after inoculation with *F. occidentalis* and for leaf trichome density

Traits	Marker at QTL peak	Chromosome	Position^a^	LOD^b^	Additive effect^c^	Dominance effect	% Expl.^d^
Damage	HpmsE078	P06	108.4	20.6	−0.66	0.10	43.9
Survival to L_2_^e^	HpmsE078	P06	108.4	24.3	−0.37	0.09	49.4
Survival to pre-pupa^f^	HpmsE078	P06	108.4	18.8	−0.34	0.10	41.1
Trichome density early vegetative^g^	HpmsE031	P10b	40.5	15.4	−0.63	0.14	30.4
Trichome density late vegetative^g^	HpmsE031	P10b	40.5	21.7	−0.69	0.26	39.9
Trichome density reproductive^g^	HpmsE031	P10b	40.5	27.5	−0.74	0.30	47.5

^a^Position of the QTL, in cM, referred to the linkage group

^b^Logarithm of the odds (LOD); for all six traits a threshold of 3.6, corresponding to a genome-wide confidence level of 0.05, was estimated from permutation tests

^c^Negative values indicate that *C. annuum* alleles result in lower genotypic values than *C. chinense* alleles

^d^Percentage of phenotypic variance explained by each QTL

^e^arcsine(sqrt(*x*)) of fraction of L_1_ larvae that survived to L_2_ stage

^f^arcsine(sqrt(*x*)) of fraction of L_1_ larvae that survived to pre-pupa stage

^g^based on a visual scale: 0 (<50 cm^−2^), 1 (50–100/cm^−2^), 2 (100–200 cm^−2^), and 3 (>200 cm^−2^) at the region near to the veins and midrib on the abaxial leaf surface of fully developed leaves at three different plant stages: early vegetative stage (3 weeks after planting), vegetative stage (6 weeks after planting), and reproduction stage (9 weeks after planting)

**Fig. 3:**
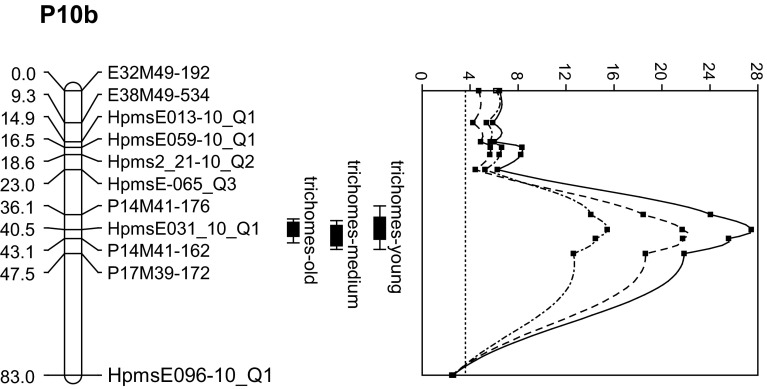
LOD profiles and 1-LOD and 2-LOD support intervals for QTL for trichome density on chromosome 10. *Solid*, *dashes*, and *dot-dashed lines* represent the trichome density at early vegetative, late vegetative, and reproductive stage, respectively. The *dotted line* at LOD 3.6 represents the LOD threshold

For each of the three leaf ages in which observations were made for leaf trichome density a highly significant QTL was detected on chromosome 10 (Fig. 3). The LOD scores for the detected QTL at all leaf ages were above the LOD score corresponding to a genome-wide confidence level of 95 %, which was 3.6 as determined by permutation test with 1000 iterations. The peak of the LOD profile for leaves at early vegetative, vegetative, and reproductive stage was near marker HpmsE031; at this marker, 30.4, 39.9, and 47.5 % of the variance of the F_2_ plant means were explained by the QTL, respectively. Use of HpmsE031 as co-factor in MQM analysis failed to uncover any additional QTLs.

### Confirmation of the resistance QTL on chromosome 6 in F_3_ lines

The effect of the QTL was validated in a set of four F_3_ lines, each originating from an F_2_ plant heterozygous at the SNP marker Isotig18917-234, close to the QTL maximum (Table 5). Overall regression analysis showed significant effects of the marker on all three parameters: damage score (*P* = 0.013), survival to L_2_ stage (*P* = 0.011), and survival to pre-pupa stage (*P* = 0.012). The effects of the individual F_3_ lines were not significantly different from each other at *P* = 0.05, except marginally between lines 40 and 43 for survival to L_2_ stage (*P* = 0.043).

**Table 5:** Mean scores for three resistance-related traits in the F_3_-line experiment, averaged per line and per genotype for marker Isotig18917-234

Trait	Damage				Survival to L_2_^a^				Survival to pre-pupa^a^			
Genotype^b^	RR	RS	SS	Total	RR	RS	SS	Total	RR	RS	SS	Total
F_3_-line												
40	0.99 (7)^c^	0.75 (5)	2.05 (5)	1.23 (17)	0.26 (7)	0.09 (5)	0.85 (5)	0.37 (17)	0.17 (7)	0.07 (5)	0.71 (5)	0.27 (17)
43	2.00 (1)	2.00 (7)	2.03 (3)	2.01 (11)	0.67 (1)	0.80 (7)	0.80 (3)	0.79 (11)	0.50 (1)	0.60 (7)	0.67 (3)	0.61 (11)
134	0.50 (1)	2.07 (5)	(0)	1.81 (6)	0.12 (1)	0.59 (5)	(0)	0.50 (6)	0.01 (1)	0.39 (5)	(0)	0.30 (6)
211	1.06 (4)	1.30 (2)	3.00 (1)	1.41 (7)	0.30 (4)	0.31 (2)	0.97 (1)	0.42 (7)	0.16 (4)	0.11 (2)	0.89 (1)	0.24 (7)
Total	1.05 (13)	1.61 (19)	2.15 (9)	1.55 (41)	0.29 (13)	0.50 (19)	0.85 (9)	0.52 (41)	0.17 (13)	0.33 (19)	0.72 (9)	0.36 (41)

^a^The means for survival to L_2_ and pre-pupa stages were calculated on the transformed scale and back-transformed to the original scale

^b^The genotypes for marker Isotig18917-234 are coded as RR: homozygous for the resistant (*C. annuum*) parent allele, RS: heterozygous, SS: homozygous for the susceptible (*C. chinense*) parent allele

^c^Numbers in brackets are the numbers of plants per category

## Discussion

### Resistance test

The high heritabilities found for thrips resistance as measured by damage, survival to L_2_, and survival to pre-pupa indicate that variation due to environmental factors was minor relative to genetic effects. This was achieved using a climate room with controlled environmental conditions and a thrips rearing that supplied us with large quantities of uniform and synchronized larvae. This is an important advantage for genetic studies in comparison with greenhouse or field tests. In previous work (Maharijaya et al. 2011), we have shown that the resistance estimated from the laboratory test corresponds well with that estimated from greenhouse and field tests.

The high correlations between damage caused by larvae, survival to L_2_, and survival to pre-pupa indicate that differences in tolerance (i.e., the ability of the plant to restrict symptom development in spite of the presence and activities of the pest) do not play an important role in this case. The low number of larvae that survived on resistant plants shows that the mechanism of pepper defense against thrips larvae is based on antibiosis (Horber 1980). It has been reported before that resistance in pepper blocks larval development of *F. occidentalis* in pepper (Maris et al. 2004; Maharijaya et al. 2012).

### Trichome density is not related to thrips resistance in pepper

No correlation was found between trichome density and any of the resistance parameters in our study with *F. occidentalis.* Also the resistant parent of our mapping population was glabrous, while the susceptible parent carried trichomes. This contrasts with an earlier finding that trichomes are associated with resistance to a different thrips species (*S*. *dorsalis*) in pepper (Yadwad et al. 2008). This difference might be caused by the difference in thrips species, but also by the fact that Yadwad et al. (2008) rated the resistance based on damage caused by adult thrips in a preference test, whereas we used a no-choice test with larvae. Further, the significant correlations of thrips resistance and trichome density found by Yadwad et al. (2008) were F_2_ population specific. For only four out of seven F_2_ populations, each consisting of 60 plants, they found a significant correlation of resistance against thrips with trichome density at the mature pepper stage (*R* = 0.27–0.48), and no correlation was found for any of those seven populations at flowering stage.

### Linkage map

Twenty-two linkage groups were constructed, for twelve chromosomes in the haploid pepper genome. The mapping of SSR markers in our linkage map was consistent with that in previous populations (Minamiyama et al. 2006; Yi et al. 2006; Barchi et al. 2007; Wu et al. 2009). The total length of our linkage map was 1630 cM which is comparable to the maps published by these authors. Although in several cases we still have more than one linkage group per chromosome, it is likely that our map covers most of the pepper genome.

### One major QTL for thrips resistance on chromosome 6 of pepper

Since the three parameters of resistance in our test: damage, survival to L_2_, and survival to pre-pupa were highly correlated (Table 3), it is not surprising that the QTLs found for those three parameters co-localize near the same markers on chromosome 6. Only one QTL was detected for all three parameters, even when using a MQM approach. This QTL explained about 50 % of the genetic variation for the three parameters, leaving the other half unexplained. Since most of the genome is covered by our linkage map the missing genetic effect cannot be caused by other major QTLs, as these would have been detected by the MQM mapping. Therefore it is likely that several QTLs with small effects are segregating in this population as well. In principle the presence of other QTL might be deduced from differences in the average level of resistance of the four F_3_ lines. We did not detect any significant F_3_-line effects, but this may be due to the limited size of each line and the fact that each segregated for the major QTL.

While the major QTL has a small dominance effect with susceptibility partially dominant over resistance, the mean of the F_2_ population is near to the midparent value and the F_1_ is more resistant than the midparent, which suggests that the residual genetic effects are (partially) dominant for resistance.

The major QTL described by Linders et al. (2010) on chromosome 5 was not detected in our study, in spite of the fact that we included several markers linked to it. Likewise they gave no hint of a possible resistance QTL on chromosome 6. As they used the same resistant parent as we did (*C. annuum* AC 1979), but a different susceptible parent, this suggests that at least two major factors are involved in the resistance present in the shared parent, but that in both mapping populations only one of these segregated. If this is true, our susceptible parent contains the resistant allele of the QTL on chromosome 5. As this parent is indeed highly susceptible (Maharijaya et al. 2011, 2012), the chromosome 5 QTL then does not provide any resistance in absence of the resistance allele on chromosome 6 QTL, and the reverse is also likely to be the case.

It is less likely that the two QTL are specific to certain subpopulations of *F. occidentalis* since the resistance donor was even resistant to two different thrips species (*F. occidentalis* and *T. parvispinus*). An alternative hypothesis is that the different QTL are due to different test methods, as the assay of Linders et al. (2010) involved a period of 3–4 weeks of population development in a choice situation, whereas we studied survival and development of individual larvae in a no-choice situation. However, in earlier experiments (Maharijaya et al. 2011), we observed no antixenosis, so the choice vs non-choice contrast is unlikely to be an explanation for the discrepancy; and if the QTL on chromosome 6 was segregating in their population it would have had a large impact on population development and most likely have been detected.

A highly significant QTL for trichome density was detected on chromosome 10. As expected from the absence of correlation between trichome density and resistance parameters, this QTL was not linked with the QTL for resistance. Our QTL for trichome density was found at the same position as the QTL found by Kim et al. (2010).

The QTL on chromosome 6 is an important factor affecting thrips resistance in pepper, which implies that pepper breeders can benefit from the introgression of this QTL. As the source of resistance is an accession of *C. annuum,* which is the dominant pepper crop species, it may be assumed that the introgression of this region to other *C. annuum* will be straightforward.

#### Author contribution statement

AM, BV, and REV conceived the project. AM performed most of the practical work and was the main author of the manuscript. BV and REV supervised the work on a daily basis and contributed extensively to the manuscript. REV also produced the linkage map and performed the QTL analyses. RGFV and AP contributed to the writing of the manuscript. GSB assisted with the insect rearing and KP assisted with the marker assays.
